# Long-Term Outcomes and Conditional Recurrence-Free Survival in Stage II Colon Cancer: The Impact of Surveillance and Recurrence Detection Strategies

**DOI:** 10.3390/jcm15134901

**Published:** 2026-06-24

**Authors:** Mustafa Alperen Tunç, Ali Kaan Güren, Burak Paçacı, Fırat Akagündüz, Erkam Kocaaslan, Ahmet Demirel, Yeşim Ağyol, Pınar Erel, Nargiz Majidova, Nadiye Sever, Naz Tayyar Tunç, Nazım Can Demircan, Selver Işık, Abdussamed Çelebi, Ezgi Çoban, Osman Köstek, İbrahim Vedat Bayoğlu, Murat Sarı

**Affiliations:** 1 Division of Medical Oncology, Department of Internal Medicine, School of Medicine, Marmara University, Istanbul 34854, Türkiye; alikaanguren@gmail.com (A.K.G.); drpacaci@gmail.com (B.P.); fratakagunduz0@gmail.com (F.A.); erkamkocaaslan@gmail.com (E.K.); drahmetdemirel23@gmail.com (A.D.); yesimagyol@gmail.com (Y.A.); pnarerell@gmail.com (P.E.); ncdemircan@gmail.com (N.C.D.); dr-selver83@hotmail.com (S.I.); abdussametcelebi@gmail.com (A.Ç.); ezgi.yuzugullu@gmail.com (E.Ç.); osmankostek@yahoo.com (O.K.); dr.vebay@gmail.com (İ.V.B.); 2 Department of Medical Oncology, VM Medical Park Maltepe Hospital, Istanbul 34846, Türkiye; nergiz.mecidova1991@gmail.com; 3 Department of Medical Oncology, Haydarpasa Numune Training and Research Hospital, Istanbul 34668, Türkiye; dr.nadya@hotmail.com; 4 Department of General Surgery, Haydarpasa Numune Training and Research Hospital, Istanbul 34668, Türkiye; naz.tayyar@sbu.edu.tr

**Keywords:** colonic neoplasms, lymph node excision, recurrence, survival analysis, chemotherapy, adjuvant, follow-up studies

## Abstract

**Background:** Adjuvant therapy decisions for T3N0 stage II colon cancer remain controversial. This study evaluates long-term outcomes, recurrence patterns, and conditional relapse-free survival (RFS) in pathologic T3N0 colon cancer. **Methods:** This retrospective study included 306 patients undergoing curative resection for T3N0 colonic adenocarcinoma (1995–2020). Early recurrence was defined as recurrence or death within 3 years after surgery. Survival was estimated via Kaplan–Meier. Cox regression, adjusted for treatment eras, evaluated survival factors. Inverse Probability of Treatment Weighting (IPTW) minimized selection bias. Conditional RFS utilized a 5-year landmark analysis. **Results:** Over a 133-month median follow-up, 72 patients (23.5%) recurred. Most recurrences (81.9%) occurred within 3 years; only 9.7% after 5 years. Five- and 10-year OS rates were 80.9% and 70.4%. Inadequate lymph node dissection (<12 nodes) was performed in 29.7% of the entire cohort and was found to be an independent adverse prognostic factor for OS. Adjuvant chemotherapy lacked overall OS benefit, though IPTW analysis suggested potential benefit in patients with inadequate dissection. Conditional RFS (5–10 years) for patients recurrence-free at 60 months was 95.0%. Exploratory analyses showed descriptive differences in post-relapse survival based on the clinical triggers prompting radiological evaluation (marker-triggered versus symptom-triggered presentations). **Conclusions:** T3N0 colon cancer recurrences occur predominantly within the first 3–5 years after surgery. Inadequate lymph node dissection is the primary adverse prognostic factor. Although a 5-year follow-up period appears adequate for most patients, individualized extended surveillance may be considered for selected high-risk patients. Adjuvant treatment and follow-up strategies should be tailored according to surgical quality and risk factors.

## 1. Introduction

Colorectal cancers remain a significant cause of mortality despite advances in screening and treatment [1]. In stage II colon cancer, curative surgery is the cornerstone of treatment, and the 5-year relapse-free survival ranges between 68% and 83% in patients treated with surgery alone [2,3]. In the T3N0 subgroup, decisions regarding adjuvant therapy are based on clinic and pathological risk factors. However, data on the impact of these risk factors on long-term survival and late recurrences are limited [4,5,6]. Although most recurrences occur within the first five years, both local and distant recurrences may still develop after five years [7,8]. The majority of existing long-term studies do not include stage-specific analyses and evaluate colon and rectal cancers together [9,10,11]. This study evaluates long-term follow-up outcomes, recurrence patterns, and conditional RFS analyses in patients with T3N0 stage II colon cancer, a group in which the role of adjuvant therapy remains controversial.

## 2. Materials and Methods

### 2.1. Study Design and Participants

**Figure 1:**
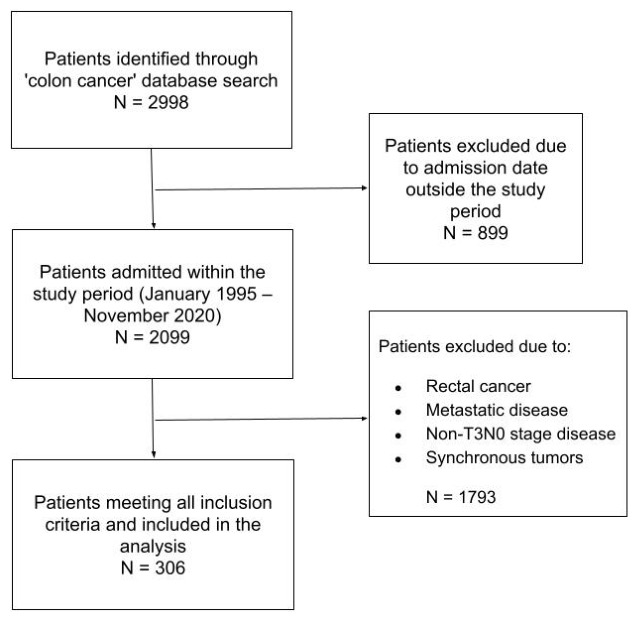
Flow diagram of patient selection.

This study was designed as a retrospective, single-center study. Patients with histopathologically confirmed colonic adenocarcinoma staged as pathological T3N0 who underwent curative surgical resection between January 1995 and November 2020 and were followed at our institution were included in the study. Patients with rectal cancer were excluded because it represents a unique clinical entity with distinct tumor biology, different recurrence patterns, and specific treatment protocols including radiotherapy; patients with stages other than T3N0, synchronous metastases, or synchronous secondary colorectal cancer were also excluded. (Figure 1).

Data were obtained from patient medical records and electronic databases. Demographic characteristics, pathological findings, adjuvant treatments, localization and dates of recurrence, dates of death, and dates of last outpatient visits were recorded. Patients were considered lost to clinical follow-up if they failed to attend scheduled surveillance visits; however, their survival status was thoroughly verified using the national electronic death registration registry. For survival analyses, patients alive at the end of the study or those lost to clinical follow-up were censored at the date of their last objective clinic contact. Tumor location was categorized as right-sided (cecum, ascending colon, hepatic flexure, transverse colon) or left-sided (splenic flexure, descending colon, sigmoid colon). To minimize potential selection bias and ensure data integrity, case screening and subsequent data extraction were performed independently by two investigators, with any discrepancies resolved by consensus. The study was approved by the Clinical Research Ethics Committee of Marmara University Faculty of Medicine (Decision No: 09.2026.26-0204). All patient data were analyzed anonymously, and the study was conducted in accordance with the Declaration of Helsinki and relevant legal regulations.

### 2.2. Follow-Up Protocol

Following curative resection, the standard follow-up strategy consisted of clinical examinations and laboratory evaluations, including serum carcinoembryonic antigen (CEA) measurements, every 3 months during the first 2 years, every 6 months between years 2 and 5, and annually thereafter. Routine surveillance imaging, including computed tomography (CT) of the chest, abdomen, and pelvis, was performed every 6 months during the first 2 years and annually thereafter. Colonoscopic surveillance was performed according to contemporary guideline recommendations and individual patient risk profiles.

### 2.3. Objectives and Definitions

The first aim of this study is to examine the factors impacting long-term survival in the follow-up of Stage II colon cancer patients. Furthermore, it seeks to determine the factors that can predict late recurrence and to assess follow-up strategies accordingly.

High-risk Stage II colon cancer was defined by the presence of at least one of the following clinicopathological risk factors: lymphovascular invasion, perineural invasion, tumor obstruction at presentation, tumor perforation, or inadequate lymph node dissection.

Inadequate lymph node dissection was defined as the retrieval of fewer than 12 lymph nodes from the surgical specimen, in accordance with international guidelines.

Relapse-free survival (RFS) was defined as the time from curative surgery to the first relapse, death from any cause, or the date of last follow-up. Overall survival (OS) was defined as the time from curative surgery to death or last follow-up. Cause-specific survival (CSS) was defined as the time from curative surgery to death directly related to colon cancer, with deaths from non-cancer-related causes treated as censored events at the time of death. Post-relapse survival (PRS) was calculated as the time from the date of first relapse to death or last follow-up.

### 2.4. Statistical Analysis

Categorical variables were presented as numbers and percentages, and continuous variables as median (IQR). Group comparisons were performed using the chi-square test or Fisher’s exact test, as appropriate. OS, RFS and PRS were estimated using the Kaplan–Meier method and compared between groups using the log-rank test. Factors associated with OS, RFS and PRS were evaluated using univariate and multivariate Cox proportional hazards regression analyses. To account for evolving standards in colorectal cancer care over the 25-year period, patients were categorized into three eras: Early (1995–2004): Pre-oxaliplatin and conventional surgery, Intermediate (2005–2012): Introduction of oxaliplatin-based adjuvant therapy and standardized Complete Mesocolic Excision (CME), Modern (2013–2020): Integration of Microsatellite Instability (MSI) testing and minimally invasive surgery. The “Era” variable was included as a covariate in multivariate models to adjust for historical bias.

The following variables were systematically analyzed in the univariate and multivariate survival models: age, gender, primary tumor location (right vs. left colon), tumor grade (well/moderate vs. poor differentiation), lymphovascular invasion, perineural invasion, tumor obstruction, tumor perforation, lymph node yield (<12 vs. ≥12), and administration of adjuvant chemotherapy.

For multivariate analysis, the threshold for clinical significance was set at *p* < 0.10. For clarity, only clinically meaningful contrasts are presented in the main manuscript, while the full models are provided in the Supplementary Materials.

In order to evaluate potential confounding by indication regarding adjuvant chemotherapy, clinicopathological characteristics were compared between patients who did and did not receive adjuvant treatment. Variables associated with receipt of adjuvant chemotherapy were subsequently evaluated using multivariable logistic regression analysis.

To minimize treatment selection bias regarding adjuvant chemotherapy, stabilized inverse probability of treatment weighting (IPTW) based on propensity scores was applied. Propensity scores were estimated using a logistic regression model including baseline clinicopathological variables considered potentially associated with treatment selection: age, gender, lymphovascular invasion, perineural invasion, mismatch repair protein (MMR) status, obstruction, and perforation. MMR status, obstruction, and perforation were included as categorical variables, with missing values retained as an “unknown” category. Stabilized weights were then generated and applied in the survival analyses. Covariate balance before and after weighting was assessed using absolute standardized mean differences (SMDs), with an SMD < 0.1 considered indicative of adequate balance.

Early recurrence was defined as recurrence or death within 3 years after surgery. Conditional RFS was estimated using a 5-year landmark analysis among patients who remained recurrence-free for 60 months. A *p*-value < 0.05 was considered statistically significant. All analyses were performed using SPSS Statistics version 25.0.

During the preparation of this manuscript, the author(s) used ChatGPT-5.5 and Gemini 1.5 Pro for the purposes of language editing, grammatical correction, literature research assistance, and structuring the introduction section. The authors have reviewed and edited the output and take full responsibility for the content of this publication.

## 3. Results

### 3.1. Patient Characteristics

A total of 306 patients with T3N0 colon cancer who underwent curative surgery between January 1995 and November 2020 were included. Demographic and pathological characteristics are presented in Table 1, and adjuvant treatments are summarized in Supplementary Table S1. The distribution of patient characteristics, surgical quality parameters (lymph node dissection), and adjuvant treatment strategies across the three chronological eras is summarized in Supplementary Table S2.

Among the 306 patients, 131 (42.8%) received adjuvant chemotherapy. Patients receiving adjuvant treatment had significantly higher rates of inadequate lymph node dissection (<12 lymph nodes examined), lymphovascular invasion, perineural invasion, obstruction, and perforation than patients who did not receive adjuvant chemotherapy (Supplementary Table S3).

To further evaluate treatment allocation, a multivariable logistic regression analysis was performed. Inadequate lymph node dissection (OR 2.76, 95% CI 1.55–4.92, *p* = 0.001), lymphovascular invasion (OR 2.88, 95% CI 1.62–5.11, *p* < 0.001), perineural invasion (OR 2.72, 95% CI 1.30–5.68, *p* = 0.008), obstruction (OR 5.49, 95% CI 2.88–10.48, *p* < 0.001), and perforation (OR 7.52, 95% CI 1.41–40.12, *p* = 0.018) were independently associated with receipt of adjuvant chemotherapy (Supplementary Table S4).

### 3.2. Survival Analyses

**Figure 2:**
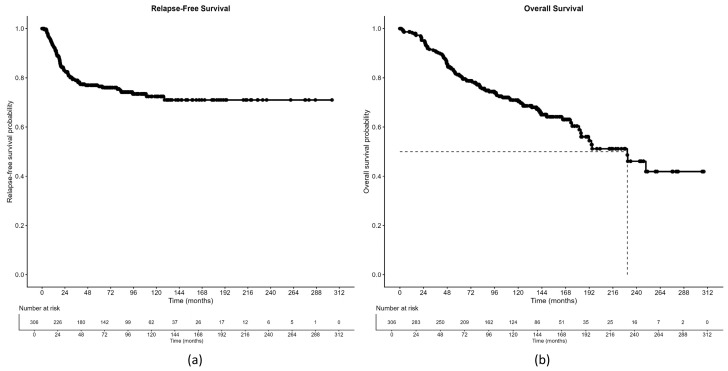
Kaplan–Meier survival curves of the study cohort (*n* = 306). (**a**) Relapse-free survival (RFS) and (**b**) Overall survival (OS) of patients with stage II (T3N0) colon cancer.

The median follow-up duration was 133 months (95% CI: 123.0–144.9). Recurrence occurred in 72 patients (23.5%). Median RFS was not reached (Figure 2a); the 5- and 10-year RFS rates were 76.5% (95% CI: 71.4–81.6) and 72.5% (95% CI: 66.8–78.2), respectively.

Patients with inadequate lymph node dissection had shorter RFS (*p* < 0.001). Presence of at least one risk factor was associated with shorter RFS (*p* = 0.006). No significant difference in RFS was observed between patients who received adjuvant chemotherapy and those who did not (*p* = 0.531) (Supplementary Table S5).

During follow-up, 102 patients (33.3%) had died. Median OS was 230 months (95% CI: 184.1–277.1) (Figure 2b), with 5- and 10-year OS rates of 80.9% (95% CI: 76.4–85.4) and 70.4% (95% CI: 64.9–75.9), respectively. Regarding cause-specific mortality, 46 deaths (45.1% of total deaths) were classified as cancer-related, while 56 (54.9%) were due to non-cancer-related causes. The 5- and 10-year Cause-Specific Survival (CSS) rates for the entire cohort were 90.8% and 84.0%, respectively. Inadequate lymph node dissection, perineural invasion, and the presence of at least one risk factor were associated with shorter OS. No significant differences in OS were observed according to sex, lymphovascular invasion, obstruction, perforation, MMR status, or receipt of adjuvant therapy (Supplementary Table S5).

To confirm the independent prognostic role of surgical quality and resolve the statistical power loss caused by model overfitting due to covariates with high missingness (such as baseline MMR status), a sensitivity analysis was performed utilizing a reduced, parsimonious multivariate Cox regression model. In this optimized model adjusted for baseline clinical characteristics, inadequate lymph node dissection remained a highly significant independent predictor of poorer survival outcomes (HR: 2.419, 95% CI: 1.447–4.047, *p* = 0.001). Additionally, primary tumor location was also identified as an independent prognostic factor, with right-sided colon tumors demonstrating a significant survival advantage over left-sided tumors in this cohort (HR: 0.544, 95% CI: 0.317–0.933, *p* = 0.027). Furthermore, formal collinearity diagnostics yielded Variance Inflation Factors (VIF) close to 1.0 across all variables, mathematically ruling out any severe multicollinearity or overlapping artifacts with chronological eras. Other covariates, including adjuvant chemotherapy, tumor obstruction, and lymphovascular invasion, did not show a statistically independent correlation with survival (Table 2).

In OS analyses, inadequate lymph node dissection was identified as an independent prognostic factor for shorter OS in both univariate and multivariate models (Table 2).

After stabilizing IPTW, covariate balance was achieved for all variables included in the propensity score model, with all absolute standardized mean differences below the predefined threshold of 0.1 (Supplementary Table S8). Adjuvant chemotherapy was not associated with improved OS in the overall cohort; however, exploratory subgroup analysis suggested improved OS among patients with inadequate lymph node dissection. IPTW-adjusted analysis confirmed a significant interaction between adjuvant chemotherapy and retrieval of fewer than 12 lymph nodes (Supplementary Figure S1).

Of the 72 patients with recurrence, 13 had local recurrence and 59 had distant metastasis. The most common site of metastasis was the liver, followed by the lung. Fifty-nine patients (81.9%) experienced recurrence within the first 3 years, while 7 patients (9.7%) developed recurrence after 5 years; only 1 patient experienced recurrence beyond 10 years. Among late recurrences, 3 patients had local recurrence and 4 had distant metastases.

When patients with recurrence were evaluated according to early versus late relapse, exploratory comparisons of baseline clinicopathological variables showed no statistically significant differences (all *p* > 0.05, Table 3). However, these analyses—particularly regarding tumor biology—must be interpreted with extreme caution. Due to the historical nature of the cohort, mismatch repair (MMR/MSI) status was evaluable in a severely limited subset of these relapsed cases (n = 20 in the early relapse group and n = 6 in the late relapse group). Consequently, while the descriptive proportion of MSI-H status was 10.0% in early and 16.7% in late recurrences (*p* = 0.654), this comparison is highly underpowered due to non-random missing data and must be treated as strictly descriptive and hypothesis-generating rather than a definitive clinical conclusion.

In the 5-year landmark analysis, conditional RFS at 5–10 years and 5–15 years was 95.0% and 93.0%, respectively. Among the seven patients who experienced recurrence after 5 years, four had inadequate lymph node dissection and five had at least one risk factor. The single patient who developed recurrence beyond 10 years presented with lung metastasis and had a left-sided colon tumor, inadequate lymph node dissection, pMMR status, and a KRAS mutation.

**Figure 3:**
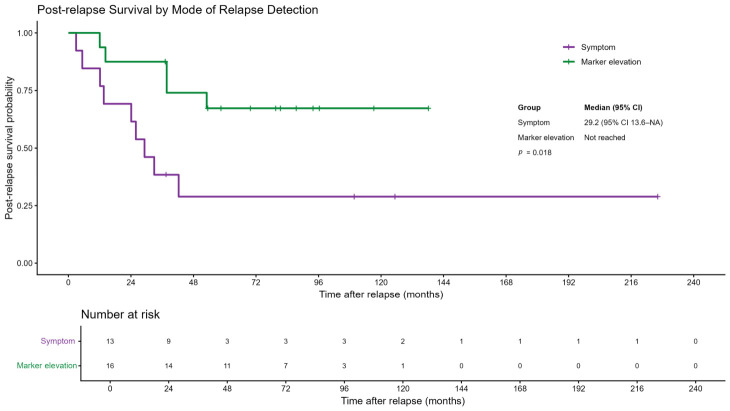
Post-relapse survival according to the mode of relapse detection. Comparison of survival outcomes among patients whose recurrences were detected via tumor markers (green line), routine surveillance imaging, or clinical symptoms (purple line). *p*-value was calculated using the log-rank test.

Among patients who developed recurrence, exploratory analyses were performed to evaluate post-relapse survival trends based on the initial clinical presentation that triggered the diagnostic workup. Crucially, to minimize chronological confounding, the official date of relapse was not defined by the initial tumor marker elevation itself; rather, for all patients, recurrence was strictly documented on the exact date that objective disease progression was radiologically confirmed on imaging. The classification into distinct groups was solely based on the primary clinical driver that prompted the diagnostic imaging evaluation (i.e., routine asymptomatic tumor marker elevation versus new onset of clinical symptoms). Kaplan–Meier analysis demonstrated a descriptive difference in post-relapse survival curves based on these triggers (log-rank *p* = 0.045), where median post-relapse survival was not reached in the marker-triggered group, compared with 29.2 months in the symptom-triggered group and 40.0 months in relapses detected during routine surveillance imaging (Figure 3). In a preliminary multivariate analysis restricted by the exploratory nature of the subsets, marker-triggered recurrence assessment was associated with a lower descriptive risk of post-relapse mortality compared with symptom-triggered presentations, while recurrence site also demonstrated a descriptive correlation (Supplementary Table S9). However, despite this standardized radiological endpoint, these descriptive differences must still be interpreted with caution as exploratory and hypothesis-generating, since retrospective comparisons remain highly sensitive to underlying tumor burden variations and the non-random missingness of historical biomarker data such as MSI status (Supplementary Table S10).

## 4. Discussion

In this study, with a median follow-up exceeding 10 years, we analyzed long-term outcomes in a homogeneous cohort limited to patients with T3N0 colon cancer. Recurrences were largely confined to the first 3–5 years after surgery, and the risk of late recurrence was very low. Inadequate lymph node dissection emerged as the most important factor affecting RFS and OS. Furthermore, exploratory assessments of the recurrence patterns revealed descriptive variations in post-relapse survival trajectories based on the initial clinical presentation that led to the diagnosis of relapse.

In our study, inadequate lymph node dissection emerged as one of the most consistent and strongest prognostic factors for RFS and OS. Previous studies have also demonstrated that inadequate lymph node dissection is associated with poor prognosis [12,13,14]. These findings further emphasize that, in stage II colon cancer, surgical quality contributes not only to accurate staging but also to long-term survival. Furthermore, our multivariate analysis revealed that although adjuvant treatment strategies and surgical standards evolved significantly across different chronological eras, the era itself did not remain an independent prognostic factor for survival. Specifically, despite the major historical transition following the FDA approval of oxaliplatin in 2002 and the subsequent updates in medical oncology protocols, these advancements did not independently dictate survival outcomes in our specific Stage II T3N0 cohort. This is clinically coherent with the understanding that routine combined oxaliplatin-based chemotherapy yields marginal benefits in low-risk Stage II disease. In contrast, the impact of adequate lymph node dissection on RFS and OS persisted regardless of the treatment period. This suggests that the survival improvements observed over the 25-year study period are primarily driven by the standardization of surgical quality rather than the temporal changes in chemotherapy regimens alone.

Randomized trials and meta-analyses have shown that intensive surveillance strategies based on imaging or carcinoembryonic antigen (CEA) monitoring do not provide a clear overall survival benefit. In contrast, observational studies have reported that recurrences detected during follow-up or in the asymptomatic phase are associated with better survival compared with symptom-detected recurrences [15,16,17]. In our study, the poorer post-relapse survival observed in patients whose recurrences were detected after symptom development suggests that tumor burden may be higher once the disease becomes clinically apparent. To ensure a standardized and objective evaluation of this survival trend, the official date of relapse in our cohort was strictly defined by radiological documentation of objective progression for all patients, rather than the timestamp of initial marker elevation. Consequently, our findings reflect the impact of the primary clinical trigger—whether an asymptomatic routine test or a symptomatic presentation—that prompted the diagnostic imaging workup. The association between earlier detection through these marker-triggered evaluations and improved post-relapse survival supports the potential clinical value of structured surveillance strategies. However, this relationship must be interpreted with extreme caution and cannot be translated into a direct causal inference, as retrospective survival intervals remain highly sensitive to underlying tumor burden variations and potential lead-time bias artifacts.

Previous studies have shown that dMMR biology is associated with prolonged RFS and represents an independent prognostic factor [18,19,20]. Although a favorable trend toward improved RFS was observed in multivariate analysis in our cohort, statistical significance was not reached. This lack of significance is directly explained by the severe data limitation at our center, where routine MMR protein analysis was not standardized before 2015, leaving only 33% of our total population (n = 101) with an evaluable MMR status. Crucially, this non-random, historically biased missingness had a profound systemic impact on our initial statistical modeling; forcing this heavily underpowered biomarker variable into the primary multivariate Cox regression caused severe model overfitting and artificially masked our core clinical predictors. As demonstrated by our subsequent sensitivity analysis, separating the MMR status from the primary parsimonious model successfully restored statistical power and allowed well-established surgical quality indicators to re-emerge with strong independent significance. Consequently, given the underpowered nature and temporal bias of our biological data, all MMR-related survival trends in Table 2 and Table 3 must be interpreted with extreme caution and treated as strictly descriptive and hypothesis-generating observations that require large-scale, prospective validation.

The role of adjuvant chemotherapy in low-risk stage II colon cancer remains controversial. Current guidelines suggest that adjuvant treatment may be considered on an individual basis in the presence of high-risk features such as inadequate lymph node dissection [2,21]. Consistent with this approach, additional analyses in our cohort demonstrated that adjuvant chemotherapy was preferentially administered to patients with recognized high-risk clinicopathological features, including inadequate lymph node dissection, lymphovascular invasion, perineural invasion, obstruction, and perforation. These findings indicate that treatment allocation largely reflected risk-adapted clinical decision-making in routine practice. Despite this selection pattern, no significant differences in RFS or OS were observed between patients who did and did not receive adjuvant chemotherapy. However, subgroup analysis suggested a potential OS benefit of adjuvant chemotherapy in patients who underwent inadequate lymph node dissection. Taken together, these findings may reflect the heterogeneous biological behavior of stage II colon cancer and the limited overall benefit of adjuvant treatment in unselected stage II populations, while supporting a potential role for adjuvant chemotherapy in carefully selected high-risk patients.

In our cohort, 81% of recurrences occurred within the first three years. The very low incidence of recurrence beyond five years indicates excellent long-term survival in patients with low-risk stage II colon cancer. Data on late recurrence are limited in the literature [22,23]. Kong et al. reported that 91.3% of recurrences in colorectal cancer occurred within the first five years; however, their cohort predominantly consisted of rectal cancer and stage III patients [11]. In the study by Frontali et al., clinicopathological characteristics of patients who remained recurrence-free for five years were evaluated, and only two patients in the colon cancer subgroup experienced recurrence after five years [10]. In our study, comparison of early and late recurrence groups suggested that a higher number of dissected lymph nodes tended to reduce the risk of early recurrence. Among patients who developed recurrence after five years, the majority had undergone inadequate lymph node dissection and had at least one additional risk factor. However, the small number of late recurrences limits causal interpretation. The finding that conditional RFS rates at 5–10 years exceeded 95% among patients who remained recurrence-free during the first 60 months highlights the very low long-term recurrence risk in this population. Based on these findings, a 5-year follow-up period appears sufficient for the majority of patients with T3N0 colon cancer. However, because late recurrences were predominantly observed among patients with inadequate lymph node dissection and additional high-risk features, selected patients may benefit from individualized surveillance strategies extending beyond the conventional 5-year period. Follow-up strategies may therefore be individualized according to the presence of risk factors.

In our study, patient surveillance relied on conventional methods including standard tumor markers, laboratory tests, and routine imaging. However, emerging artificial intelligence and deep learning algorithms are rapidly transforming colorectal cancer care by enhancing early serologic, endoscopic, and radiological screening, as well as digital histology evaluation—though their clinical integration currently awaits large-scale prospective validation. From a future perspective, incorporating these technological advancements and novel biomarkers is expected to provide significant improvements in accurate staging, early recurrence detection, and overall prognosis.

This study has several limitations. First, its retrospective design introduces inevitable selection and measurement biases related to patient selection, treatment decisions, and follow-up practices. Second, the long study period may have resulted in heterogeneity due to temporal changes in surgical techniques, pathological assessment, adjuvant treatment regimens, and surveillance strategies. Third, incomplete MMR data limited the ability to more robustly assess the prognostic impact of this biomarker. The small number of late recurrences also limited the statistical power of analyses in this subgroup. Finally, the single-center design may restrict generalizability; however, the homogeneous patient population and long follow-up duration represent important strengths.

## 5. Conclusions

In this long-term study, recurrences in patients with T3N0 stage II colon cancer were largely confined to the first 3–5 years, with a very low risk of recurrence beyond five years. The high conditional recurrence-free survival observed in patients who remained recurrence-free during the early period indicates an excellent long-term prognosis. Inadequate lymph node dissection emerged as the most important adverse prognostic factor, while adjuvant chemotherapy did not confer a significant survival benefit. These findings suggest that adjuvant treatment and surveillance strategies in T3N0 colon cancer may be planned in a more selective and individualized manner.

## Figures and Tables

**Table 1:** Baseline Patient and Pathological Characteristics.

	T3N0 *n* (306)
Age (years), median (IQR)	63 (53–72)
Gender, n (%)	
Male	177 (57.8%)
Female	129 (42.2%)
Tumour localization, n (%)	
Right	142 (46.4%)
Left	164 (53.6%)
Tumour diameter (mm), median (IQR)	50 (40–64)
Lymph node, n, median (IQR)	16 (11–23)
Inadequate lymph node dissection, n (%)	
Yes	91 (29.7%)
No	215 (70.3%)
Lymphovascular invasion, n (%)	
Yes	100 (32.7%)
No	206 (67.3%)
Perineural invasion, n (%)	
Yes	51 (16.7%)
No	255 (83.3%)
Obstruction, n (%)	
Yes	72 (23.5%)
No	227 (74.2%)
Missing	7 (2.3%)
Perforation, n (%)	
Yes	13 (4.2%)
No	286 (93.5%)
Missing	7 (2.3%)
MMR status, n (%)	
dMMR	26 (8.5%)
pMMR	75 (24.5%)
Missing	205 (67%)
K-RAS, n (%)	
Mutant	18 (5.9%)
Wild	16 (5.2%)
Missing	272 (88.9%)
N-RAS, n (%)	
Mutant	2 (0.7%)
Wild	22 (7.2%)
Missing	282 (92.2%)
BRAF, n (%)	
Mutant	1 (0.3%)
Wild	19 (6.2%)
Missing	286 (93.5%)

Abbreviations: MMR: Mismatch repair; dMMR: Deficient mismatch repair; pMMR: Proficient mismatch repair; K-RAS: Kirsten rat sarcoma virus oncogene; N-RAS: Neuroblastoma rat sarcoma virus oncogene; BRAF: B-Raf proto-oncogene; n: Number of patients.

**Table 2:** Combined Univariate and Multivariate Cox Regression Analyses for Relapse-Free Survival and Overall Survival in Patients with T3N0 Colon Cancer.

Variable	RFS Univariate HR (95% CI)	*p*	RFS Multivariate HR (95% CI)	*p*	OS Univariate HR (95% CI)	*p*	OS Multivariate HR (95% CI)	*p*
Sex	0.80 (0.50–1.27)	0.350	—	—	0.87 (0.58–1.29)	0.488	—	—
Tumor location (Right vs. Left colon)	0.40 (0.24–0.67)	0.001	0.54 (0.317–0.933)	0.027	0.79 (0.53–1.18)	0.255	—	—
Lymphovascular invasion	1.14 (0.70–1.84)	0.594	1.08 (0.652–1.814)	0.747	1.48 (0.99–2.23)	0.054	1.35 (0.87–2.08)	0.174
Perineural invasion	1.58 (0.90–2.77)	0.104	—	—	1.61 (1.00–2.60)	0.052	1.44 (0.86–2.42)	0.162
Obstruction	1.26 (0.74–2.14)	0.382	1.152 (0.648–2.049)	0.629	1.34 (0.86–2.07)	0.185	—	—
dMMR	0.37 (0.12–1.10)	0.075	—	—	0.68 (0.22–2.06)	0.504	—	—
Inadequate lymph node dissection	2.78 (1.74–4.43)	<0.001	2.41 (1.447–4.047)	<0.001	2.37 (1.60–3.51)	<0.001	2.01 (1.32–3.07)	<0.001
Adjuvant chemotherapy	1.15 (0.73–1.84)	0.532	0.89 (0.517–1.564)	0.707	0.96 (0.65–1.42)	0.852	—	—
Chronological EraΔ		0.035	Reference	0.582		0.030	Reference	0.249

Abbreviations: RFS: Relapse-free survival; OS: Overall survival; HR: Hazard ratio; CI: Confidence interval; dMMR: Deficient mismatch repair. Variables with *p* < 0.10 in univariate analysis were selected for the multivariate model. ΔMultivariate models were adjusted for the chronological era of treatment. Full hazard ratios and confidence intervals for the era analysis are presented in Supplementary Tables S6 and S7.

**Table 3:** Comparison of clinicopathological characteristics between early (≤3 years) and late (>3 years) recurrence among patients with disease recurrence.

Variable	Early Relapse ≤ 3 Years	Late Relapse > 3 Years	*p* Value
Total number of retrieved lymph nodes, median (IQR)	11 (5–17)	16 (9–25)	0.068
Inadequate lymph node dissection (<12), n (%)	31/59 (53.4)	6/13 (46.2)	0.634
MSI-H status ^†^, n (%)	2/20 (10.0)	1/6 (16.7)	0.654 *
Receipt of adjuvant therapy, n (%)	27/59 (45.8)	7/13 (53.8)	0.597

Abbreviations: IQR, Interquartile range; MSI-H, Microsatellite instability-high. ^†^ Due to the retrospective design and historical testing protocols, MMR/MSI status was evaluable in only 26 out of 72 relapsed patients (36.1% data availability). * Statistical comparison for MSI status is highly underpowered due to small, non-random historical subsets and should be interpreted strictly as descriptive and hypothesis-generating.

## Data Availability

The data supporting the findings of this study are available from the corresponding author upon reasonable request.

## Supplementary Materials

The following supporting information can be downloaded at https://www.mdpi.com/article/10.3390/jcm15134901/s1: Table S1. Adjuvant therapies received by patients, Table S2: Evolution of Surgical and Oncological Management Across Eras, Table S3: Clinicopathological Characteristics According to Receipt of Adjuvant Chemotherapy, Table S4: Multivariable Logistic Regression Analysis of Factors Associated with Receipt of Adjuvant Chemotherapy, Table S5. Kaplan–Meier Survival Comparisons for Relapse-Free Survival (RFS) and Overall Survival (OS) in T3N0 Colon Cancer, Table S6: Univariate Cox Regression Analysis for Chronological Eras, Table S7: Multivariate Cox Regression Analysis for Chronological Eras, Table S8: Covariate balance before and after stabilized IPTW, Table S9. Independent predictors of post-relapse mortality, Table S10. Multivariate Cox regression analysis for post-relapse mortality, Figure S1. Forest plot of the interaction between adjuvant chemotherapy and lymph node dissection adequacy on overall survival.

## Abbreviations

The following abbreviations are used in this manuscript:

RFS	Relapse-Free Survival
OS	Overall Survival
IPTW	Inverse Probability of Treatment Weighting
PRS	Post-Relapse Survival
CME	Complete Mesocolic Excision
MSI	Microsatellite Instability
AMD	Absolute Mean Difference
dMMR	Deficient Mismatch Repair
pMMR	Proficient Mismatch Repair
CEA	Carcinoembryonic Antigen
