# Supplementary material for: Long-Term Outcomes and Conditional Recurrence-Free Survival in Stage II Colon Cancer: The Impact of Surveillance and Recurrence Detection Strategies
Source: J Clin Med. 2026 Jun 24;15(13):4901. doi: 10.3390/jcm15134901 (PMC13362504; doi:10.3390/jcm15134901)
Supplement: Supplementary file 1 [file jcm-15-04901-s001.zip › jcm-4353511-supplementary tables.pdf]

Supplementary Table S1. Adjuvant therapies received by patients

Treatment	N:131 (100%)
**5-FU**	61 (46.6%)
**Capecitabine**	33 (25.2%)
**FOLFOX**	17 (13%)
**CAPOX**	20 (15.3%)

Abbreviations: > 5-FU: 5-fluorouracil; FOLFOX: 5-fluorouracil, leucovorin, and oxaliplatin; CAPOX: Capecitabine and oxaliplatin.

Supplementary Table S2: Evolution of Surgical and Oncological Management Across Eras

Characteristics	Early Era (1995–2004) (n=45)	Intermediate Era (2005–2012) (n=98)	Modern Era (2013–2020) (n=163)	p-value*
**Surgical Quality**				**<0.001**
>Lymph node dissection				
- Adequate, n (%)	21 (46.7%)	57 (58.2%)	137 (84.0%)	
- Inadequate, n (%)	24 (53.3%)	40 (41.8%)	26 (16.0%)	
**Adjuvant Strategy**				**<0.001**
- No Chemotherapy, n (%)	23 (51.1%)	47 (48.0%)	105 (64.4%)	
- 5-FU -based, n (%)	22 (48.9%)	37 (37.8%)	35 (21.5%)	
- Oxaliplatin-based, n (%)	0 (0.0%)	14 (14.3%)	23 (14.1%)	

Abbreviations: 5-FU: 5-fluorouracil; n: Number of patients.

Notes: *P-values were calculated using the chi-square test or Fisher's exact test to evaluate the distribution of characteristics across the three chronological eras. Adequate lymph node dissection was defined as the retrieval of ≥12 lymph nodes

Supplementary Table S3: Clinicopathological Characteristics According to Receipt of Adjuvant Chemotherapy

Variable	No Adjuvant Chemotherapy (n=175)	Adjuvant Chemotherapy (n=131)	P value
**Female sex**	74 (42.3%)	55 (42.0%)	0.958
**Male sex**	101 (57.7%)	76 (58.0%)	
**Right-sided tumor**	76 (43.4%)	66 (50.4%)	0.228
**Left-sided tumor**	99 (56.6%)	65 (49.6%)	
**Inadequate lymph node dissection (<12)**	37 (21.3%)	53 (40.5%)	**<0.001**
**Lymphovascular invasion**	37 (21.1%)	63 (48.1%)	**<0.001**
**Perineural invasion**	16 (9.1%)	35 (26.7%)	**<0.001**
**Obstruction**	19 (11.2%)	53 (41.1%)	**<0.001**
**Perforation**	4 (2.3%)	9 (7.0%)	0.082*
**MSI-H**	18 (31.0%)	9 (20.9%)	0.257

* Fisher's exact test.

Supplementary Table S4: Multivariable Logistic Regression Analysis of Factors Associated with Receipt of Adjuvant Chemotherapy

Variable	OR	95% CI	P value
**Inadequate lymph node dissection (<12)**	2.76	1.55–4.92	**0.001**
**Lymphovascular invasion**	2.88	1.62–5.11	**<0.001**
**Perineural invasion**	2.72	1.30–5.68	**0.008**
**Obstruction**	5.49	2.88–10.48	**<0.001**
**Perforation**	7.52	1.41–40.12	**0.018**

Variables entered into the multivariable logistic regression model included inadequate lymph node dissection, lymphovascular invasion, perineural invasion, obstruction, and perforation. Odds ratios (ORs) represent independent associations with receipt of adjuvant chemotherapy.

Supplementary Table S5. Kaplan–Meier Survival Comparisons for Relapse-Free Survival (RFS) and Overall Survival (OS) in T3N0 Colon Cancer

Variable	Mean RFS (months)	RFS p	Mean OS (months)	OS p
**Sex (Male/Female)**	222.1 vs 214.1	0.349	198.3 vs 198.1	0.488
**Tumor location (Right/Left colon)**	242.9 vs 200.1	**<0.001**	204.1 vs 193.7	0.254
**Adequate lymph node dissection**	250.6 vs 163.6	**<0.001**	220.6 vs 149.9	**<0.001**
**Lymphovascular invasion**	231.7 vs 183.7	0.594	210.2 vs 165.1	0.052
**Perineural invasion**	231.4 vs 175.9	0.101	206.6 vs 150.6	**0.049**
**Obstruction**	228.5 vs 201.4	0.381	208.2 vs 170.9	0.183
**Perforation**	225.9 vs 216.6	0.889	200.1 vs 187.3	0.738
**MMR status (pMMR/dMMR)**	96.2 vs 115.8	0.065	139.3 vs 128.8	0.501
**Presence of ≥1 high-risk feature**	258.0 vs 193.1	**0.006**	246.2 vs 166.3	**<0.001**
**Adjuvant chemotherapy**	232.2 vs 202.6	0.531	202.6 vs 189.1	0.852
**Chronological era (Early/Intermediate/Modern)**	227.4 / 152.5 / 125.7	**0.031**	181.6 / 159.6 / 130.6	**0.028**

Supplementary Table S6: Univariate Cox Regression Analysis for Chronological Eras

Chronological Era	RFS Hazard Ratio (95% CI)	p-value	OS Hazard Ratio (95% CI)	p-value
**Early (1995–2004)**	Reference	—	Reference	—
**Intermediate (2005–2012)**	1.39 (0.70–2.77)	0.353	0.96 (0.56–1.64)	0.880
**Modern (2013–2020)**	0.71 (0.36–1.42)	0.337	0.54 (0.31–0.97)	**0.038**
**Overall (Omnibus Test)**		**0.031**		**0.028**

Abbreviations: RFS: Relapse-free survival; OS: Overall survival; HR: Hazard ratio; CI: Confidence interval.

Notes: *P-values were calculated using Univariate Cox Proportional Hazards Regression. The Early Era (1995–2004) was used as the reference group for all comparisons. The Omnibus test p-value indicates the overall significance of the chronological era as a categorical variable.

Supplementary Table S7: Multivariate Cox Regression Analysis for Chronological Eras

Chronological Era	RFS Multivariate HR (95% CI)	p-value	OS Multivariate HR (95% CI)	p-value
**Early (1995–2004)**	Reference	—	Reference	—
**Intermediate (2005–2012)**	1.88 (0.20–17.78)	0.582	0.99 (0.58–1.69)	0.880
**Modern (2013–2020)**	—	—	0.67 (0.37–1.22)	0.190
**Overall (ERA)**		0.582		0.249

Abbreviations: RFS: Relapse-free survival; OS: Overall survival; HR: Hazard ratio; CI: Confidence interval.

Notes: *P-values were calculated using Univariate Cox Proportional Hazards Regression. The Early Era (1995–2004) was used as the reference group for all comparisons. The Omnibus test p-value indicates the overall significance of the chronological era as a categorical variable.

Supplementary Table S8: Covariate balance before and after stabilized IPTW

Covariate	SMD before IPTW	SMD after IPTW
**Age**	0.388	0.012
**Gender**	0.005	0.019
**Lymphovascular invasion**	0.268	0.014
**Perineural invasion**	0.175	0.013
**MMR proficient**	0.030	0.002
**MMR deficient**	0.035	0.009
**MMR unknown**	0.005	0.011
**Obstruction: No**	0.288	0.016
**Obstruction: Unknown**	0.013	0.005
**Obstruction: Yes**	0.301	0.011
**Perforation: No**	0.051	0.016
**Perforation: Unknown**	0.000	0.002
**Perforation: Yes**	0.051	0.014

Supplementary Table S9. Independent predictors of post-relapse mortality

Variable	Adjusted HR (95% CI)	p
Marker-detected vs symptom-detected recurrence	0.05 (0.005–0.55)	**0.014**
Recurrence site	8.81 (1.04–74.95)	**0.046**

***Routine imaging-detected recurrence was not significantly associated with post-relapse mortality and is not shown for clarity. Abbreviations: HR: Hazard ratio; CI: Confidence interval.**

Notes: *P-values were calculated using Multivariate Cox Proportional Hazards Regression. Symptom-detected recurrence was used as the reference group for the detection method comparisons. A p-value < 0.05 was considered statistically significant.

Supplementary Table S10. Multivariate Cox regression analysis for post-relapse mortality

Variable	Adjusted HR	95% CI	p
**Marker vs symptom**	0.05	0.005–0.55	**0.014**
**Routine vs symptom**	0.21	0.03–1.43	0.111
**Recurrence site**	8.81	1.04–74.95	**0.046**
**MSI status**	4.23	0.65–27.45	0.131

Abbreviations: HR: Hazard ratio; CI: Confidence interval; MSI: Microsatellite instability.

Notes: *P-values were calculated using Multivariate Cox Proportional Hazards Regression. Symptom-detected recurrence was used as the reference group for the detection method comparisons. A p-value < 0.05 was considered statistically significant.
