# Supplementary figures and images for: Long-Term Outcomes and Conditional Recurrence-Free Survival in Stage II Colon Cancer: The Impact of Surveillance and Recurrence Detection Strategies
Source: J Clin Med. 2026 Jun 24;15(13):4901. doi: 10.3390/jcm15134901 (PMC13362504; doi:10.3390/jcm15134901)

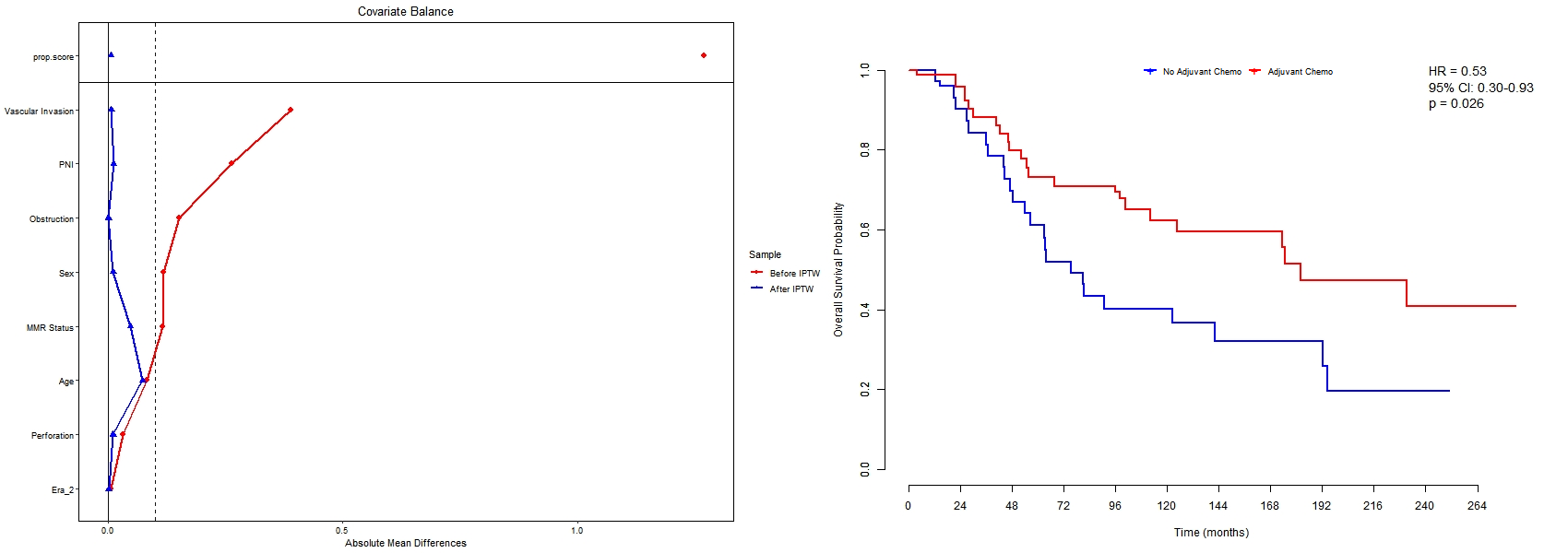

Supplement: Supplementary file 1 [file jcm-15-04901-s001.zip › Supplementary Figure S1.tiff]
